# A New Generation of X-ray Baggage Scanners Based on a Different Physical Principle

**DOI:** 10.3390/ma4101846

**Published:** 2011-10-17

**Authors:** Konstantin Ignatyev, Peter R.T. Munro, Deeph Chana, Robert D. Speller, Alessandro Olivo

**Affiliations:** 1 Department of Medical Physics and Bioengineering, UCL, Gower Street, London WC1E 6BT, UK; E-Mails: k.ignatyev@medphys.ucl.ac.uk (K.I.); pmunro@medphys.ucl.ac.uk (P.R.T.M.); rspeller@medphys.ucl.ac.uk (R.D.S.); 2 Department for Transport, Victoria Street, London SW1E 6DT, UK; E-Mail: Deeph.Chana@dft.gsi.gov.uk

**Keywords:** baggage scanners, x-ray refraction, x-ray phase, phase contrast imaging

## Abstract

X-ray baggage scanners play a basic role in the protection of airports, customs, and other strategically important buildings and infrastructures. The current technology of baggage scanners is based on x-ray attenuation, meaning that the detection of threat objects relies on how various objects differently attenuate the x-ray beams going through them. This capability is enhanced by the use of dual-energy x-ray scanners, which make the determination of the x-ray attenuation characteristics of a material more precise by taking images with different x-ray spectra, and combining the information appropriately. However, this still has limitations whenever objects with similar attenuation characteristics have to be distinguished. We describe an alternative approach based on a different x-ray interaction phenomenon, x-ray refraction. Refraction is a familiar phenomenon in visible light (e.g., what makes a straw half immersed in a glass of water appear bent), which also takes place in the x-ray regime, only causing deviations at much smaller angles. Typically, these deviations occur at the boundaries of all objects. We have developed a system that, like other “phase contrast” based instruments, is capable of detecting such deviations, and therefore of creating precise images of the contours of all objects. This complements the material-related information provided by x-ray attenuation, and helps contextualizing the nature of the individual objects, therefore resulting in an increase of both sensitivity (increased detection rate) and specificity (reduced rate of false positives) of baggage scanners.

## 1. Introduction

X-ray scanners are an essential component in the protection of vital infrastructure as they are used to detect threat objects (explosives, weapons, *etc.*) and prevent their introduction into strategically important buildings. Although other inspection methods exist (terahertz, ultrasound, *etc.*), x-rays are routinely used to scan baggage, parcels or even cargo.

Overall, x-ray attenuation-based baggage scanning is an effective and reliable technique; however, as it will be described below, it suffers from limitations in sensitivity (detection rate) and specificity (rate of false positives). We discuss here a different approach which results in increasing both its sensitivity and specificity: x-ray phase contrast imaging (XPCi).

### Basic Principles and Limitations of Attenuation-based X-ray Baggage Scanning

**Figure 1:**
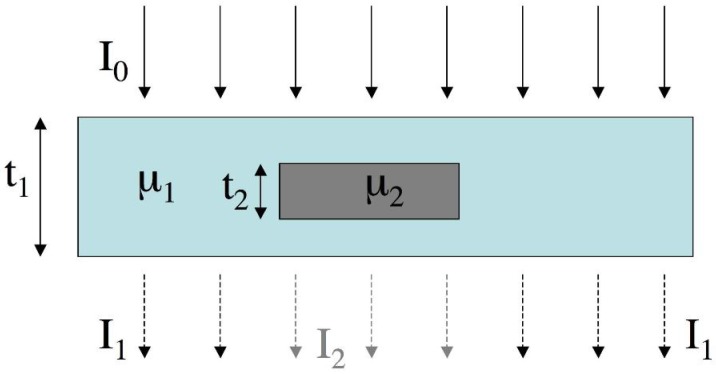
Working principle of conventional, attenuation-based x-ray imaging. The detail must stop (or deviate at an angle sufficiently large to take them out of the original beam) more (or less) x-rays than the surrounding background in order to be detected.

To date, almost all commercially available x-ray imaging systems are based on x-ray attenuation. This exploits the difference between the attenuation properties of the targeted detail and that of the surrounding background to generate image contrast (see Figure 1). The attenuation properties are expressed by means of the material’s *attenuation coefficient* μ.

The attenuation contrast of the detail is defined as:

(1) $$C_{att}=\frac{I_{1}-I_{2}}{I_{1}}$$

where *I*_2_ and *I*_1_ are the x-ray intensities in the shadow of the detail and in the surrounding area, respectively. Assuming that an x-ray intensity given by *I*_0_ impinges on the detail, that the attenuation coefficient of the detail and of the background are *μ*_2_ and *μ*_1_ respectively, that the overall thickness of the object is *t*_1_ and the thickness of the detail *t*_2_, by using the Beer/Lambert law [1] we can write:

(2) *I*_1_ = *I*_0_ exp(−*μ*_1_*t*_1_)

and

(3) *I*_2_ = *I*_0_ exp[−*μ*_1_(*t*_1_ − *t*_2_)]exp(−*μ*_2_*t*_2_)

and therefore, replacing (2) and (3) into Equation (1):

(4) *C_att_* = 1 − exp[(*μ*_1_ − *μ*_2_]*t*_2_]

Formally, these equations only hold for monochromatic x-ray beams. In practice, this is never the case in x-ray baggage scanners, and the equations have to be integrated over the x-ray spectrum as the attenuation coefficient *μ* is a function of energy (which explains why, as mentioned in the abstract, dual-energy methods are often used to allow the attenuation coefficient to be estimated—see for example ref. [2]). However, the simplified model leading to Equation (4) allows us to underline the following:

- if the thickness of the imaged object (*t*_2_) is small, *C_att_* tends to zero;
- if the attenuation coefficients of detail (*μ*_2_) and background (*μ*_1_) are very similar (*i.e.*, the term *μ*_1_ – *μ*_2_ is small), *C_att_* tends to zero.

These are the main limitations of conventional, attenuation based x-ray imaging, and, as underlined by the simplified model, they are intrinsic to the contrast formation mechanisms and therefore inescapable as long as x-ray attenuation is used.

In addition to this, there is another complication that can create problems in security scans. Even in the case where *μ*_2_ is sufficiently different from *μ*_1_, and therefore a good image contrast is obtained, it may be difficult to identify the material the detail is made of, *i.e.*, to distinguish it from other materials with a similar attenuation coefficient. For example, there is a range of materials with an attenuation coefficient similar to that of explosives, such as other plastics, cheeses, some liquids, *etc.* It would be very useful in this case to be able to identify other characteristic features, like thin wires forming part of a detonator device, which would enable contextualizing the plastic object therefore reducing the rate of false positives (*i.e.*, increased specificity). Unfortunately, as these wires are often thin, they can be missed in conventional inspections, as they might not produce sufficient image contrast (small *t*_2_, *i.e.*, item “a” above).

The above summarizes the origins of the sensitivity and specificity limitations of current, attenuation-based baggage scanners, and provides a typical example where improvement in such areas would be needed. In the following, we will describe a different approach, and show how this could be used to remove some of these limitations.

## 2. X-ray Phase Contrast Imaging (XPCi)

Alongside attenuation, which is the combination of x-ray absorption and scattering at angles sufficiently large to remove the x-rays from the primary beam, other phenomena take place when x-rays transverse a material. These phenomena, which are linked to the phase changes that x-rays undergo when crossing an object, are normally discarded in conventional x-ray imaging. In order to understand the nature of these phenomena, it is important to remember that, although in the “attenuation” picture x-rays are basically treated like particles, they are actually electromagnetic waves. Waves take different speeds when they travel through different materials, and, as a consequence, waves which were initially in phase may not be when they exit a given detail. The basic principle of XPCi is to exploit these phenomena to generate image contrast.

Attenuation-based methods only deal with changes in intensity, which, as discussed above, can be insufficient to generate adequate contrast in a number of situations. Changes of phase, unconsidered by conventional approaches, can however generate intense image contrast, as they can result in interference effects: waves with different phases interfere with each other constructively and destructively, creating strong intensity minima and maxima. In other words, even if the overall energy transported by the wave is not changed (e.g., in the case of negligible attenuation and therefore of no intensity variation), the spatial distribution of energy can change substantially, leading to intense minima and maxima in the detected signal. These can reveal features of the samples that created them.

A simplified but effective way to treat this class of phenomena is to rely on the refractive index. This can be written as [3]:

(5) *n* = 1- *δ* + *iβ*

where *i* is the imaginary unit. The imaginary term *β* is responsible for x-ray attenuation: using a semi-classical approximation, it is in fact possible to write [4]:

(6) $$\mu =\frac{2\pi \beta }{\lambda }$$

where *λ* is the x-ray wavelength.

The difference from unity of the real part, *δ*, is responsible for the phase shift that the x-ray wavefront undergoes when crossing an object. Despite being several order of magnitudes greater than *β* for most materials in the x-ray energy range [5], in conventional x-ray imaging methods *δ* is completely unconsidered. The fact that *δ* >> *β*, however, means that a much higher image contrast can be obtained if changes in *δ* are properly exploited.

**Figure 2:**
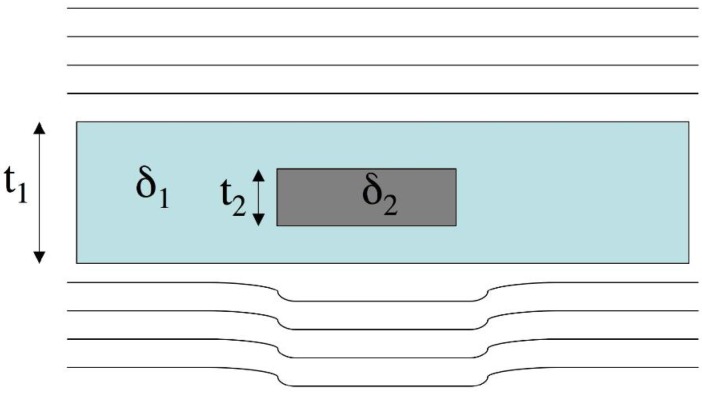
Working principle of phase contrast imaging. An initially perfectly plane wavefront, with all waves “in phase”, becomes distorted because waves have a higher speed inside the detail than outside (see text).

Figure 2 replicates the scheme of Figure 1, but this time with the x-rays seen as waves. For simplicity’s sake, let us neglect variations in *β* (as it can be done in cases where *C_att_* is small), and characterize the background and the detail by means of their *δ* value. For simplicity’s sake, a plane wave impinging on the sample is considered, but the use of a spherical wave would not change the essence of what is discussed below: it would only change the shape of the wavefront and introduce a degree of magnification, as discussed by Wilkins *et al.* [6]. The case in which the presence of the detail advances the wavefront is represented (as it would happen by placing an homogeneous object in air, for example), but even when the opposite happens the substance of what is discussed below does not change.

It should be noted that the overall x-ray intensity might not have been changed at all by the presence of the object (this would be the case for a “pure” phase object, *i.e.*, with *β*_1_ = *β*_2_ = 0 and both *δ*_1_ and *δ*_2_ ≠ 0), or a decrease in the overall intensity might have occurred, but the x-ray intensity in the shadow of the detail and outside is the same (*β*_1_ = *β*_2_ ≠ 0), and therefore *C_att_* = 0. In both these cases, the object would be invisible to conventional, attenuation-based x-ray imaging, but could be detected by XPCi thanks to the wavefront distortion resulting from having *δ*_1_ ≠ *δ*_2_. Likewise, also in cases where there is a moderate but non-zero attenuation contrast (*β*_1_ and *β*_2_ slightly different), the difference between *δ*_1_ and *δ*_2_ can be used to enhance the detail visibility. The next section will present ways to translate this wavefront distortion into image contrast.

### 2.1. Different Approaches to XPCi

The simplest implementation of XPCi is free-space propagation (FSP). By allowing the x-rays enough space to propagate beyond the sample, the anticipated (or delayed) and non-anticipated (or non-delayed) portions of the wavefront will interfere with each other, resulting in the generation of an interference pattern [7] *i.e.*, a series of positive and negative peaks in the detected x-ray intensity. The problem with this approach is that, for a detectable interference pattern to be produced, the x-ray source must exhibit a high degree of spatial coherence, *i.e.*, the source size (the focal spot, for a conventional x-ray tube) must be small and/or seen from a long distance [3]. This is summarized by the concept of projected source size *s_p_*:

(7) $$s_{p}=\frac{d_{sd}}{d_{ss}}s$$

where s is the real source size (in the direction transverse to x-ray propagation), and *d_sd_* and *d_ss_* are the sample-to-detector and the source-to-sample distances, respectively.

**Figure 3:**
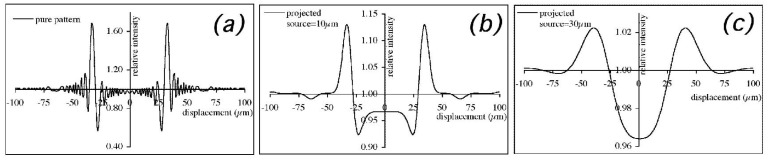
The effect of an increasing projected source size s_p_ on the FSP phase contrast pattern. The pattern in (**a**) was created by numerically solving the Fresnel/Kirchoff diffraction integral [3] for a 50 µm thick polyethylene wire, and convolved with Gaussian distributions with a width of 10 and 30 µm to obtain plots (**b**) and (**c**), respectively.

An example is provided in Figure 3. Figure 3(a) shows the “pure” FSP pattern that would be achieved with an ideal point source (practically never achievable in real cases), while Figure 3(b,c) show the effect of a projected source size equal to 10 μm and 30 μm, respectively. The position of the highest peaks corresponds to the edges of the imaged detail. The important aspect to note is that the vertical scale has been drastically changed in order to accommodate the three plots in the same figure. It should also be noted that, given the substantial difference between the signals shown in Figure 3 and attenuation-type signals, in this case it is convenient to introduce a “peak-to-peak” contrast:

(8) $$C_{XPCi}=\frac{I_{\max}-I_{\min}}{I_{background}}$$

where *I*_max_ is the maximum intensity of the positive fringes, *I*_min_ is the minimum intensity of the negative ones, and *I_background_* is the background intensity outside the imaged detail. The plots of Figure 3 were normalized to *I_background_*, so that *C_XPCi_* can be directly read-out on the vertical axis, leading to *C_XPCi_* values equal to approximately 90%, 20% and 7% for the point source, the 10 μm projected source and the 30 μm projected source, respectively. This demonstrated the decrease of FSP phase contrast with the increase in the projected source size (and consequently also with the increase in the real source size).

As a consequence of this, FSP produces satisfying results only with synchrotron [7,8] or micro-focal x-ray sources [6]. When *s_p_* approaches values of the order of ~100 μm, XPCi-related image enhancement tend to vanish [8], and therefore when conventional, non-microfocal sources are used, phase effects become very difficult to appreciate in the images [9]. Alongside explaining why XPCi effects are not normally detected by conventional x-ray imaging systems (together with the fact that the detector is usually positioned immediately behind the sample, and the x-rays are not given enough space to propagate), this also explains why FSP-based XPCi cannot be directly translated into real-world applications requiring fast throughput. Synchrotrons are clearly out of the question, and the low power associated with micro-focal sources means that exposure times which could be as long as hours [6] are required.

**Figure 4:**
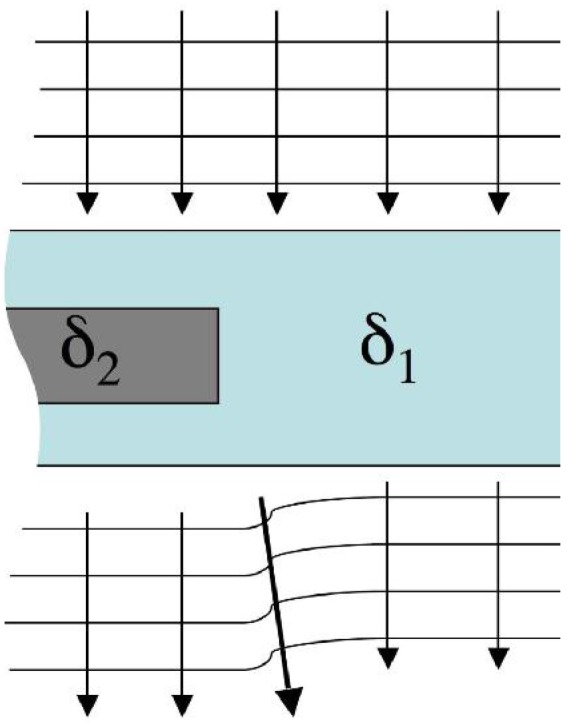
Interpretation of wavefront distortions in terms of changes in the x-ray direction.

In order to enable such a translation, we followed a different approach to convert wavefront distortions into image contrast. This is schematized in Figure 4, which shows a portion of the schematic shown in Figure 2 in which the x-ray directions are highlighted by arrows. According to the ray-tracing approximation [3], the direction of the x-rays is locally orthogonal to the wavefront. This means that, to a good approximation, the x-rays still travel in their original directions behind the bulk of the detail and outside it, but this is not the case in the vicinity of the detail edges, where the wavefront is most distorted (as underlined by the thick arrow under the detail in Figure 4), resulting in a detectable difference in the x-ray direction. It should be noted that the effect has been highly exaggerated in the figure for clarity’s sake, and that those deviations are of the order of tens of microradians (~1/1,000 of a degree).

This is basically the phenomenon of x-ray refraction. The ray-tracing approximation enables calculating the deviation angle as:

(9) $$\alpha =\frac{\lambda }{2\pi }\left|{\vec{\nabla }}_{x,y}\Phi \right|=\left|{\vec{\nabla }}_{x,y}\left\lfloor \int_{object}\delta (x,y,z)dz\right\rfloor \right|$$

where *z* is the direction along which the x-rays are propagating. Φ is the total phase shift, calculated as the integral of *δ* extended over the entire thickness of the imaged object along the *z* direction, multiplied by 2*π*/*λ* (for a homogeneous object, the integral is simply *δ* multiplied by the object thickness). ${\vec{\nabla }}_{x,y}$ is the gradient operator where derivatives are performed along the transverse directions only. This means that the deviation angle depends on the spatial variations of *δ* times the object thickness: the sharper these variations (e.g., at the edge of a detail), the larger the deviation of the x-rays transversing that region.

Therefore, by devising a method sensitive enough to detect these deviations in the x-ray direction, image contrast can be generated which arises at the interfaces between different materials. For what said above, the sharper are the transition between materials with different *δ* values, the easier is the detection of the effect or, equivalently, the stronger is the contrast that can be obtained with a system with a given angular sensitivity.

A very sensitive method is obtained by exploiting the reflectivity curves of perfect crystals [10]. However, the use of perfect crystals requires the beam to be parallel and monochromatic, and therefore once again restricts the method to synchrotron radiation environments. In principle, crystals can be used with conventional sources, but they would automatically select the appropriate parallel and monochromatic components, which consist of only a small fraction of the total flux emitted by the source. This means that the source is used very inefficiently, and the exposure times are consequently long.

We have devised a different method, which achieves a comparable directional selectivity while employing a fully divergent, polychromatic beam generated by a conventional source [11].

**Figure 5:**
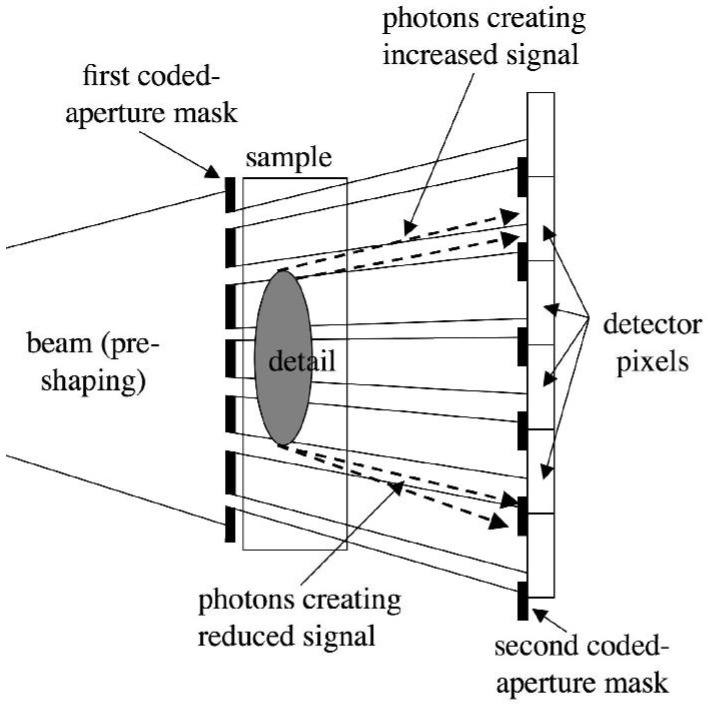
Schematization of the coded-aperture XPCi method.

The method employs two sets of aperture masks. One is placed immediately before the imaged sample, and splits the beam into a plurality of individual beams. The second is placed in contact with the detector, and creates insensitive regions between adjacent pixels. The two masks are identical apart from a scaling factor which accounts for the beam divergence, and are positioned in such a way that each individual beam created by the first mask straddles the edge of one of the apertures in the second mask. This is schematized in Figure 5.

The fact that the beams created by the first mask fall on the edge between sensitive and insensitive regions of the detector (e.g., in absence of the sample, 50% of the x-rays in each beam would be detected), means that a very small deviation of these beams (or of parts of the beams) will generate a change in the number of x-rays detected by each pixel. An example of this is shown in Figure 5. The upper edge of the detail inside the sample will deviate some x-rays upwards. As a consequence of this, x-rays which would, in absence of the sample, hit the solid parts of the detector mask, and therefore not be detected, are deviated into one of the mask apertures. Thus, they will reach the detector and be counted. This translates into an increase in the number of counts detected by the involved detector pixels, and therefore into a bright fringe appearing along the upper edge of the detail. The opposite occurs at the bottom edge of the detail. In this case, photons are sent downwards and deviated from one of the apertures (which they would have hit in absence of the sample) into the solid part of the mask. As a consequence, a reduction in the number of counts is observed in the corresponding pixels, and a dark fringe will appear along the lower border of the detail. Therefore, an x-ray transparent detail will become visible because of intense bright and dark fringes appearing along its edges. This produces images similar to those obtained using crystals [12], but the use of an aperture mask instead of a crystal means that divergent and polychromatic beams, like those emitted by conventional x-ray sources, can be used. Some practical examples in which objects producing insufficient x-ray attenuation are detected because of these fringes will be shown below.

### 2.2. Experimental Set-up

A conventional, rotating anode tungsten source previously used in hospital work since the mid-eighties [13] was used in this work. It features a source size of the order of 50 μm, about one order of magnitude larger than sources previously used in XPCi work outside synchrotrons. The Hamamatsu C9732DK passive-pixel CMOS flat panel (Hamamatsu, Japan) was used as detector device. The pre-sample and detector coded-aperture masks were built to the authors’ design by Creatv Microtech (Potomac, MD), and consisted of long parallel slits carved on a 150 μm thick gold mask electroplated on a graphite substrate. The area of the detector mask was 6 × 6 cm^2^, while the pre-sample mask was scaled down to 4.8 × 4.8 cm^2^ to account for the beam divergence, as the distances from the source to the pre-sample and detector masks were of 1.6 m and 2.0 m, respectively. The relatively small mask dimensions are due to the proof-of-concept nature of the present work, and it is possible to scale the system up to dimensions compatible with real baggage scanners. The aperture pitch was 100 μm in the detector mask, and scaled down to 80 μm in the sample mask again to account for the divergent nature of the beam. The parallel apertures were as long as the masks in one direction, and their number was sufficient to cover the masks completely along the other (*i.e.*, 6 cm/100 μm = 600 parallel apertures in total). In this case, the aperture dimension was 1/3 of the pitch for both masks. This was chosen in order to be able to experiment with dark field imaging approaches (*i.e.*, the condition in which the masks are arranged in such a way that they block off the primary beam completely) alongside the XPCi approach described above. In a dedicated system, a fill-factor of 50% would be chosen instead, in order to allow more photons through the mask thus reducing the exposure time. The masks were mounted on multiple-axis alignment stages consisting of Newport (Irvine, CA) Microcontrole translators and Kohzu (Kurigi, Japan) cradles. These were used first to align the apertures in the detector mask with the detector pixel columns, and then to align the pre-sample mask with the detector one. All the above components were mounted on a Melles-Griot (Albuquerque, NM) optical breadboard without vibration damping and/or insulation. This was done intentionally, to verify that a normal level of environmental vibrations could be accepted without excessively affecting image quality.

**Figure 6:**
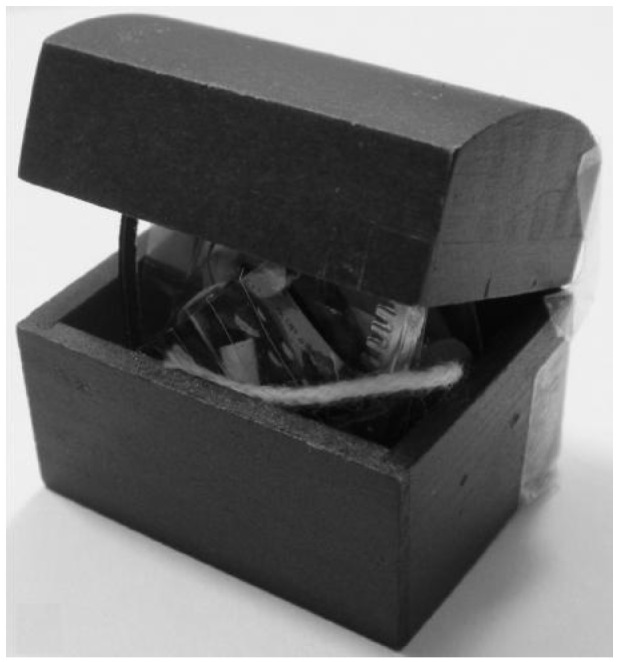
One of the samples used in the proof-of-concept experiment.

Two samples were built by hiding a small plastic cylinder, mimicking the explosive, and thin aluminum and polyethylene wires, mimicking detonator components in a toy wooden chest (see Figure 6) and in a toy purse. Additional objects were progressively added to simulate increasingly cluttered conditions. Both samples were imaged both with the XPCi method described above and in the conventional attenuation modality. The latter was achieved by removing both coded-aperture masks, placing the sample in contact with the detector, and reducing the x-ray tube current to compensate for the x-rays stopped by the mask. This was done in order to have the same photon flux reaching the detector, and therefore the same x-ray statistics in the images (*i.e.*, the same noise level).

## 3. Results and Discussion

**Figure 7:**
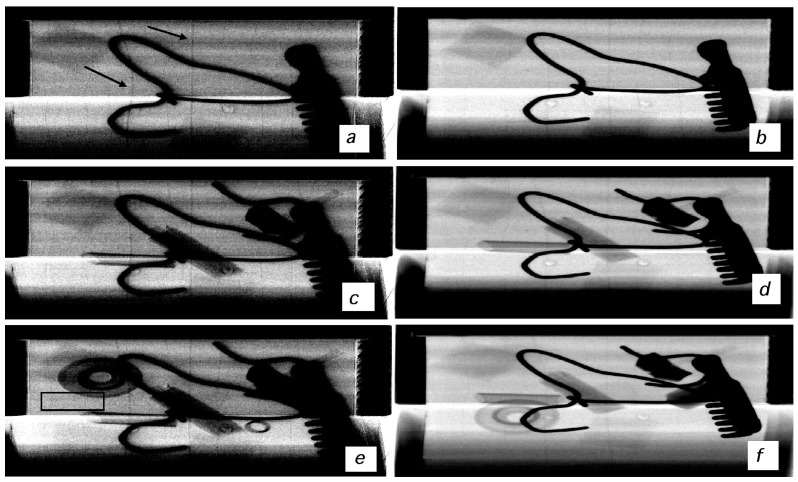
XPCi (left-hand side) *vs.* conventional (right-hand side) images of one of the custom test objects. The level of clutter is increased going from top to bottom. Arrows in (**a**) highlight the presence of two of the “thicker” (300 µm) wires: the position of these and other wires is the same for all other images (see text). The box in (**e**) highlights the area expanded in Figure 8(a); exactly the same area was extracted from (**f**) to obtain the image shown in Figure 8(b).

The first batch of images of the wooden chest is presented in Figure 7. Coded-aperture XPCi images are shown on the left hand side (Figure 7(a,c,e) while the attenuation-based images are shown on the right hand side (Figure 7(b,d,f). Going from the images at the top to the images at the bottom of the figure, progressively more objects were added to increase the level of clutter. Although such objects did not always end up in exactly the same position, the same objects were introduced in corresponding XPCi and conventional images (a and b, c and d, *etc.*). Two types of objects are detected in the XPCi images while they are invisible in the conventional, attenuation-based ones: 75 μm thick aluminum wires, and 300 μm thick polyethylene fibers. The former simulate thin electrical conductors which could form part of a detonator device, while the latter could be binding fibers and/or insulator coatings of the electrical wires. It is important to notice that, although the “cluttering” objects have moved from one image to the other, the wires were fixed to the internal chest walls and as a consequence are always located in the same position.

**Figure 8:**
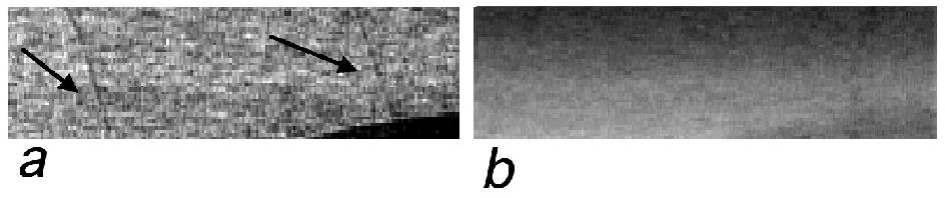
Blow-up region extracted from Figure 7(e) (see rectangle in Figure 7(e)) showing the visibility of the thin aluminum wires in the XPCi images (**a**). The same area was extracted from Figure 7(f) (**b**), showing that in this case the wires are invisible. Note that the border of the disk-shaped object, visible in the bottom right corner, is also enhanced by XPCi.

Increased detectability can be observed in all XPCi images, regardless of the level of clutter. In fact, if the cluttering object is not fully absorbing therefore stopping all the x-rays, the wires and fibers can still be seen “behind” the object itself. It should also be noted that the cluttering objects themselves, unless when fully absorbing, are better visualized in the XPCi images, which would lead to easier identification of all objects in a bag/suitcase if needed.

The 300 μm polyethylene fibers can be easily spotted in the images on the left hand side, while seeing the 75 μm aluminum wires could prove more difficult. This is primarily because of the huge disproportion between the size of the aluminum wires and that of the chest itself, which is almost as big as the coded-aperture masks. Figure 8 shows a blown up region taken from Figure 7(e), in the vicinity of the cluttering object shaped like a disk (top-left corner of Figure 7(e)), to demonstrate that also the aluminum wires exhibit a strong contrast. It should be noted that this contrast arises primarily in the form of intense dark and bright fringes at either side of the detail, underlying its phase nature. In fact, both the aluminum and polyethylene details are practically invisible in the attenuation-based images on the right hand side of Figure 7, with only the thicker polyethylene details producing a faint shadow easily disguised by the cluttering objects.

**Figure 9:**
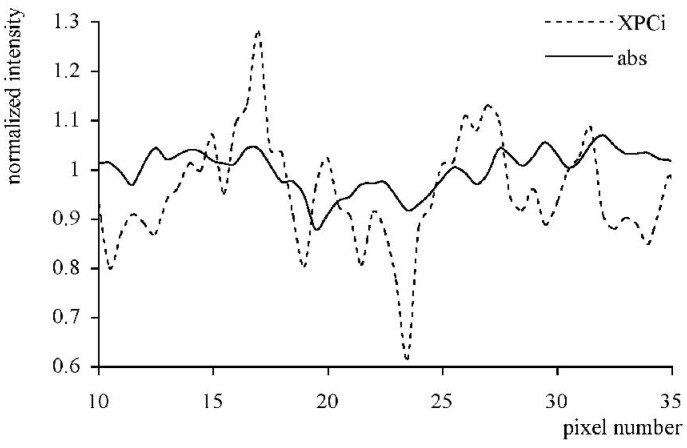
Profiles of the 300 µm polyethylene wire extracted from the XPCi (dashed line) and from the absorption (solid line) images of Figure 7(a,b) respectively. The profile corresponds roughly to the position indicated by the top arrow in Figure 7.

Figure 9 shows an image profile extracted from one of the 300 µm polyethylene wires, for both the phase contrast and the absorption images. Each profile was divided by the overall number of counts in the background (*i.e.*, immediately outside the wire itself), so that relative intensity variations (*i.e.*, the contrast) can immediately be read-out on the vertical axis. The presence of the wire is immediately visible in the XPCi profile (dashed line) due to the strong positive and negative peaks (at pixel positions equal to approximately 17 and 24) typical of differential phase contrast profiles. This shows an impressive peak-to-peak contrast of almost 70% for a weak absorbing sample, which demonstrates the high sensitivity of the coded-aperture XPCi method. Conversely, just a small reduction in intensity can be observed at the corresponding wire positions for the solid line, representing the absorption signal. It should be noted that the “noisier” profile of the XPCi signal is due to the fact that we are shining x-rays through wood: the high sensitivity of the XPCi method makes the structure of the wood visible, creating some sort of “structural” noise. This notwithstanding, the highly increased contrast leads to a much higher signal-to-noise ratio and infact the wires are only visible in the XPCi images.

**Figure 10:**
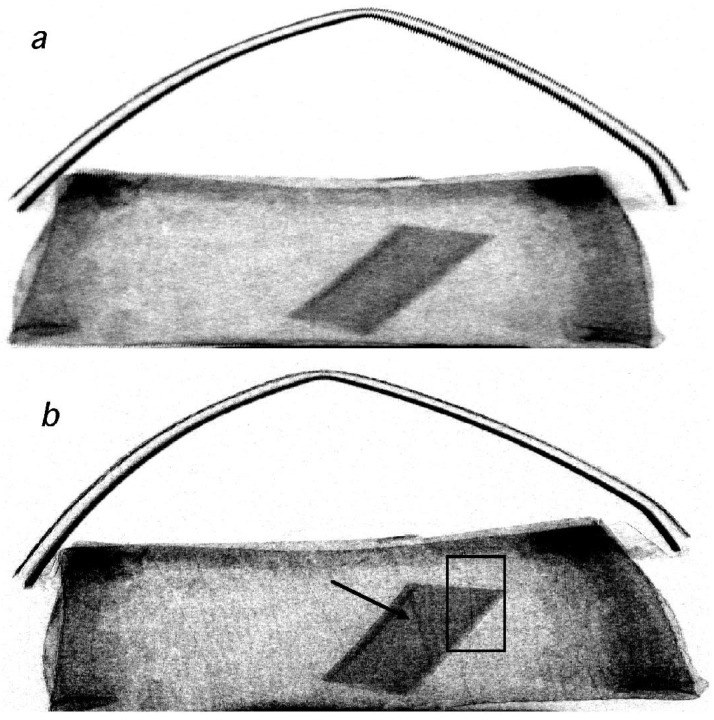
XPCi (**b**) *vs.* conventional (**a**) images of the second custom test object. An arrow underlines the presence of the thick (300 µm) wire. The area used to obtain the image in Figure 10 is highlighted by a rectangle.

Another example was obtained by imaging a toy purse, containing a plastic cylinder (the simulated “explosive”) and thin wires. This is shown in Figure 10, where again XPCi (b) and attenuation-based (a) images are compared.

In this case, an extra level of complication was intentionally added. The purse is filled with synthetic wool, which consists of a huge number of microscopic fibers tangled together. Each one of these fibers creates x-ray refraction, thus sending x-rays in all possible directions therefore disturbing the phase contrast effect. However, they cannot be individually resolved as they are well below the resolution limit of the used detector. This can be appreciated by noting the “speckled” appearance of Figure 9(b), as noted by other authors dealing with a similar problem on other samples [14]. Despite this, substantial advantages are still obtained over the conventional attenuation method. The 300 μm polyethylene fiber (only one was present in this case) is visible above, behind and below the plastic cylinder representing the explosive in Figure 10(b), while it can hardly be seen in Figure 10(a). The 75 μm aluminum wires are again detectable in Figure 10(b) and not in Figure 10(a), albeit with an overall reduced contrast compared to the previous case of the chest (it should be noted, however, that also the wooden structure of the chest disturbs the phase contrast to some extent, while seeing the wires through a uniform material would result in a much higher contrast [15]).

**Figure 11:**
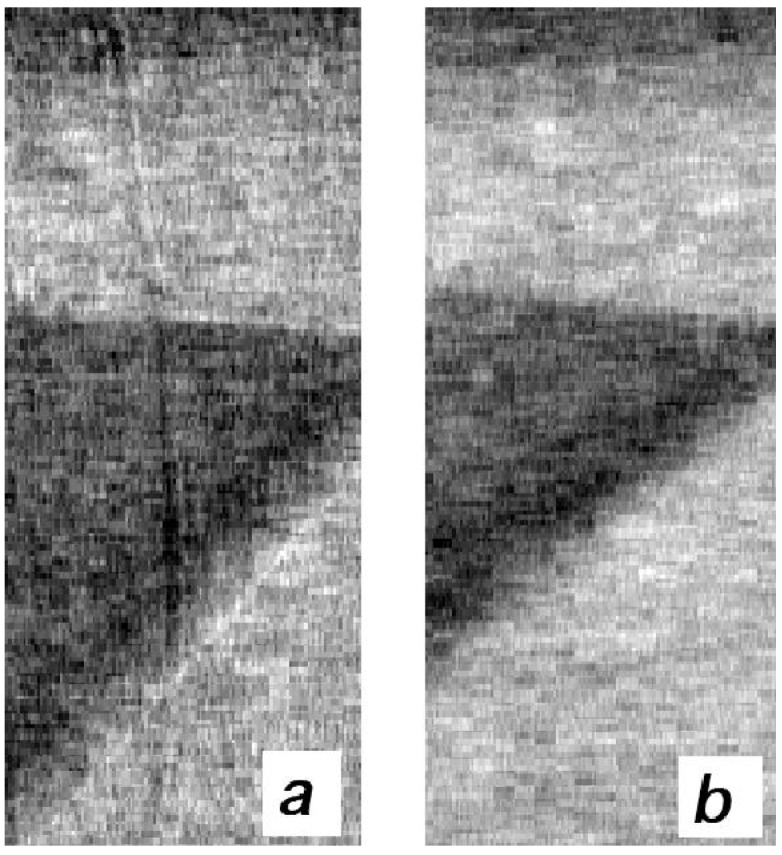
Blown-up region taken across the top-right corner of the plastic cylinder visible in Figure 10 (see rectangle in Figure 10b) showing how the 75 μm aluminum wires are detected in the XPCi (**a**) but not in the attenuation-based (**b**) image. Note that in (a) the wire is still visible also behind the cylinder itself (NB here (a) is XPCi and (b) is absorption).

Like in the previous case, seeing these thin wires in Figure 10 might be difficult because of scaling issues, but they can clearly be appreciated in the blown-up region shown in Figure 11.

## 4. Conclusions

A new x-ray imaging method, based on the detection of phase effects, was presented and its improved detail detection capability over conventional, attenuation-based x-ray imaging demonstrated on two custom-built phantoms mimicking cases of interest in security scans. Even though at present a system with a field of view of only 6 × 6 cm^2^ was built to prove the concept, the technology is easily scalable to much larger fields of view. Not only was our prototype system built with components available off the shelf, but some possible improvements (more modern x-ray sources with higher output, vibration damping, *etc.*) were intentionally left out to demonstrate the method’s robustness. In order to avoid damaging the target, all acquisitions were performed with a maximum tube current of 1 mA (at 80 kVp), which resulted in sub-minute exposure times. Sources currently available on the market which allow tube currents of up to 45 mA (e.g., Rigaku—see www.rigaku.com) would enable exposure times of the order of one second, thus compatible with the requirements of security scans. The method can be easily implemented both in a stationary acquisition modality with an area detector, or in a scanned-acquisition mode with a strip detector, as is more often done in security inspections. We expect that, following the successful outcome of this proof-of-concept study, a full-scale prototype will be built which would enable the translation of the technology into use in real-world applications, and ultimately its deployment at customs and airports.

Finally, it should be noted that the use of XPCi and dual-energy methods are not mutually exclusive. Dual-energy methods can be easily implemented in FSP by means of an energy-resolved detector [16], although, as said above, FSP is hard to implement with conventional sources when short exposure times are required. Recently, a method to combine XPCi and Dual Energy was proposed for grating interferometry techniques [17], by designing an interferometer in which different Talbot orders result in the same Talbot distance for different energies. A similar implementation would be much simpler in the approach proposed here, as the method is completely achromatic and allows reaching higher x-ray energies, as discussed in [18].

## References

[1] Johns H.E., Cunningham J.R. (1983). The Physics of Radiology.

[2] Webb S. (1988). The Physics of Medical Imaging.

[3] Born M., Wolf E. (1980). Principles of Optics.

[4] Raven C., Snigirev A., Snigireva I., Spanne P., Souvorov A, Kohn V. (1996). Phase-contrast microtomography with coherent high-energy synchrotron x-rays. Appl. Phys. Lett..

[5] Henke B.L., Gullikson E.M., Davis J.C. (1993). X-ray interactions: Photoabsorption, scattering, transmission and reflection at E = 50–30,000 eV, Z = 1–92. At. Data Nucl. Tables.

[6] Wilkins S.W., Gureyev T.E., Gao D., Pogany A., Stevenson A.W. (1996). Phase-contrast imaging using polychromatic hard x-rays. Nature.

[7] Snigirev A., Snigireva I., Kohn V., Kuznetsov S., Schelokov I. (1995). On the possibilities of x-ray phase contrast microimaging by coherent high-energy synchrotron radiation. Rev. Sci. Instrum..

[8] Arfelli F., Assante M., Bonvicini V., Bravin A., Cantatore G., Castelli E., Dalla Palma L., di Michiel M., Longo R., Olivo A. (1998). Low-dose phase contrast x-ray medical imaging. Phys. Med. Biol..

[9] Kotre C.J., Birch I.P. (1999). Phase contrast enhancement of x-ray mammography: A design study. Phys. Med. Biol..

[10] Chapman D., Thomlinson W., Johnson R.E., Washburn D., Pisano E., Gmur N., Zhong Z., Menk R., Arfelli F., Sayers D. (1997). Diffraction-enhanced x-ray imaging. Phys. Med. Biol..

[11] Olivo A., Speller R. (2007). A coded-aperture technique allowing x-ray phase contrast imaging with conventional sources. Appl. Phys. Lett..

[12] Olivo A., Arfelli F., Cantatore G., Longo R., Menk R.H., Pani S., Prest M., Poropat P., Rigon L., Tromba G. (2001). An innovative digital imaging set-up allowing a low-dose approach to phase contrast applications in the medical field. Med. Phys..

[13] Buckland-Wright J.C. (1989). A new high-definition microfocal x-ray unit. Br. J. Radiol..

[14] Kitchen M.J., Paganin D., Lewis R.A., Yagi N., Useugi K., Mudie S.T. (2004). On the origin of speckle in x-ray phase contrast images of lung tissue. Phys. Med. Biol..

[15] Olivo A., Ignatyev K., Munro P.R.T., Speller R.D. (2011). Non-interferometric phase contrast imaging with incoherent x-ray sources. Appl. Opt..

[16] Olivo A., Speller R. (2006). Experimental validation of a simple model capable of predicting the phase contrast imaging capabilities of any x-ray imaging system. Phys. Med. Biol..

[17] Kottler C., Revol V., Kaufmann R., Urban C. (2010). Dual energy phase contrast x-ray imaging with Talbot-Lau interferometer. J. Appl. Phys..

[18] Ignatyev K., Munro P.R.T., Chana D., Speller R.D., Olivo A. (2011). Coded apertures allow high-energy x-ray phase contrast imaging with laboratory sources. J. Appl. Phys..

